# Antioxidant and Anti-Proliferative Effects of *Ornithogalum balansae* Extracts Collected in Black Sea Region Against Lung Cancer Cells

**DOI:** 10.3390/life14111365

**Published:** 2024-10-24

**Authors:** Nebahat Ejder, Munevver Sökmen, Fatma Tunalı, Sevgi Kolaylı, Ali Osman Kılıç

**Affiliations:** 1 Department of Medical Microbiology, Faculty of Medicine, Recep Tayyip Erdoğan University, 53100 Rize, Türkiye; 2 Department of Bioengineering, Konya Food and Agriculture University, 42080 Konya, Türkiye; munevversokmen@gmail.com (M.S.);; 3 Department of Chemistry, Faculty of Sciences, Karadeniz Technical University, 61080 Trabzon, Türkiye; skolayli@ktu.edu.tr; 4 Department of Medical Microbiology, Faculty of Medicine, Karadeniz Technical University, 61080 Trabzon, Türkiye

**Keywords:** *Ornithogalum balansae*, anti-proliferative, antioxidant, lung cancer cells, migration, invasion

## Abstract

*Ornithogalum* is a genus of wild herb species widely used as food and in traditional medicine. This study investigated some of the biologically active properties of *Ornithogalum balansae* grown in the eastern Black Sea region of Türkiye. The investigations were carried out using the methanolic extracts of the plant’s aerial and bulbs (B1 and B2) parts. The phenolic composition was examined as total phenolic content (TPC), total flavonoid content (TFC), and HPLC-PDA. The antioxidant capacity of the extracts was tested based on ferric reducing/antioxidant power (FRAP) and 2,2-diphenyl-1-picrylhydrazyl (DPPH) radical scavenging methods. The anti-proliferative activity was tested against metastatic cell lines H1299 and H209, non-small-cell lung A549, and fibroblast MRC-5 cell lines using MTT and trypan blue methods. Wound-healing and invasion chamber assays were used to determine the inhibitory effects of the extracts on migration and invasion, respectively. The extract of the aerial part contained a large number of phenolic substances and high antioxidant capacity. The extract exhibited a significant anti-proliferative effect on the human lung cancer cells (A549 and H209), with IC_50_ values of 0.97 ± 0.04 and 1.06 ± 0.07 µg/mL, respectively. Moreover, the aerial part exhibited inhibition of migratory and invasive capacities in A549 cells at a concentration of 1.50 µg/mL. The findings associated with *O. balansae* suggest a promising therapeutic potential against lung cancer cells.

## 1. Introduction

The genus *Ornithogalum*, a member of the family Liliaceae, consists of approximately 70 species widely distributed in Türkiye [1]. Historical records dating as far back as Dioscorides (40–90 BC) document the use of *Ornithogalum* species for culinary and medicinal purposes, particularly against abscesses and as an emetic [2]. In the eastern Black Sea region of Türkiye, the aerial parts and bulbs of *Ornithogalum* are traditionally employed as a food source [3,4]. Several species within the genus *Ornithogalum* exhibit diverse biological activities, including anti-inflammatory and anti-tumor properties [5,6]. In vitro and in vivo investigations have elucidated the anti-tumoral effects of *Ornithogalum* species across various cancer types and have explored potential therapeutic targets [7,8,9,10]. Notably, some steroidal glycosides, isolated from *Ornithogalum saundersiae*, have demonstrated significant anti-proliferative effects against A549 lung adenocarcinoma cells. Its potent anti-tumoral activity, surpassing that of calprotectin, doxorubicin, and paclitaxel, identifies it as a potential candidate for anti-cancer drug development. Therefore, studies on new drugs and treatment approaches have gained momentum in recent years [10].

Lung cancer remains the foremost cause of cancer-related mortality worldwide, contributing to an estimated 2.2 million new cases and 1.8 million deaths annually [11,12]. Its aggressive nature, tendency to metastasize, and resistance to conventional anti-cancer therapies pose significant challenges in terms of patient management. Metastatic lung cancer exhibits a propensity to spread to various organs, including the lymph nodes, liver, bones, brain, and adrenal glands, severely limiting the effectiveness of treatment [13]. Moreover, existing treatments such as conventional chemotherapy and radiation therapy are not only costly but also frequently result in adverse effects on healthy tissues [14,15]. The exploration of novel therapeutic modalities has thus gained traction in recent years.

One such innovative approach is the investigation of new plant-derived natural compounds, known for their potential anti-cancer properties. Various plant species are reservoirs of bioactive compounds with promising anti-cancer activity. In our prior research, we documented the inhibitory effects of extracts obtained from *Ornithogalum balansae* bulb and flower on four cancer cell lines (A549, HT-29, CRL-2923, and HeLa), revealing a notably selective inhibitory impact on A549 cells [16]. Building upon that research, the present study centered on the extraction of the bulb and aerial part compounds from *O. balansae*, a species indigenous to the Rize (Ovit), Türkiye region, using maceration extraction techniques. These extracts were subsequently evaluated for their anti-tumor properties against normal fibroblast (MRC-5), non-small-cell lung cancer (A549), and metastatic lung cancer cell lines (H209 and H1299). 

Additionally, the study aimed to explore the antioxidant activity of *O. balansae* extracts while demonstrating their in vitro anti-proliferative effects on lung cell lines via scratch wound and invasion assays. In this study, the display of biological activity efficacy of *O. balansae* extracts would lead to the purification of new anti-cancer extracts and form the basis for new drug design studies.

## 2. Materials and Methods

### 2.1. Materials

Dulbecco phosphate-buffered saline (D-PBS), RPMI-1640, Dulbecco’s Modified Eagle’s Medium (DMEM), and Iscove’s Modified Dulbecco’s Medium (IMDM) were purchased from Capricorn (Düsseldorf, Germany). Dimethyl sulfoxide (DMSO), 3-(4,5-dimethylthiazol-2-yl)-2,5-diphenyltetrazolium bromide (MTT), and trypan blue were obtained from Sigma-Aldrich (St. Louis, MO, USA). Fetal bovine serum (FBS), penicillin-streptomycin (10,000 U/mL), and trypsin-EDTA were purchased from Gibco (Carlsbad, CA, USA). QCM EC Matrix Cell Invasion Assay, 24-well (8 µm), colorimetric was purchased from Merck (Darmstadt, Germany). Taxol was purchased from Serva (Heidelberg, Germany).

### 2.2. Cell Culture 

The A549 and H1299 cell lines were provided by Prof. Dr. Fikrettin Şahin of Yeditepe University and Associate Prof. Huri Dedeakayoğullari from Istinye University Istanbul, Türkiye. H209 and MRC-5 were purchased from the Foot and Mouth Disease Institute in Ankara, Türkiye. Cells were cultured in RPMI-1640 or DMEM or IMDM medium supplemented with 10% FBS and 1% penicillin-streptomycin and incubated in a 5% CO_2_ humidified atmosphere at 37 °C (Thermo Fisher, Waltham, MA, USA).

### 2.3. Plant Material

The plant specimens were gathered from the Ovit-Rize, Türkiye mountains within the Black Sea region of Türkiye in June 2022. Following harvesting, the bulbs and aerial parts were meticulously separated from the fresh plants and subsequently dried in a Memmert UF 55 oven until a stabilized dry weight was achieved at 50 °C.

### 2.4. Extraction Procedure

The methanolic extracts were obtained from the dried plant samples via maceration. For this process, 3 g of powdered sample was combined with 30 mL of methanol in a 50 mL Falcon tube. The mixture was agitated on a shaker for 48 h at ambient temperature. The extracts were then filtered through filter paper, and the solvent was removed by evaporation with a Rotary evaporator (IKA^®^-Werke, RV 05 Basic, Staufen, Germany). Subsequently, the aerial parts and bulb (Bulb1) extracts were dissolved in DMSO. The bulb (Bulb2) was also dissolved in water (Sigma-Aldrich, W1503, USA), designated as B1 and B2, and stored at −20 °C. 

### 2.5. Morphological Studies

The cell lines included three lung cancer cells—A549, H1299, and H209—and one fibroblast cell, MRC-5. These were cultured in their respective media, stained with trypan blue, and counted using a hemocytometer. Cell suspensions were dispensed at 4 × 10^4^ cells/mL of 12-well microplates and incubated for 48 h. At the end of the incubation, the medium was replaced with fresh medium. The negative controls (medium alone and medium with 0.1% DMSO) and positive controls including aerial and bulb extracts were prepared in the media at the desired concentrations and added to the cells. Morphological changes occurring in the cells were examined after 48 h using an inverted microscope (Olympus CKX41, 4X, Tokyo, Japan), and the results were photographed.

### 2.6. MTT Assay

The cells were allowed to adhere for 48 h until confluence, treated with the extract at concentrations from 50 to 0.78 µg/mL, and incubated for 48 h at 37 °C. The new medium was then added and treated with 100 µL of MTT solution (5 mg/mL) for 4 h at 37 °C. Absorbance was detected at 570 nm using a microplate reader (Thermo Scientific Multiskan GO, Waltham, MA, USA). The results were calculated as IC_50_ (µg/mL). The IC_50_ value is the amount of extract that reduces the cells in the medium by half.

### 2.7. Trypan Blue Assay

The A549 and H209 cells (4 × 10^4^ cells/mL) were seeded into 12-well culture plates in 1 mL of growth medium in triplicate. Following overnight incubation, various concentrations of the extracts (12 to 1.50 µg/mL) or solely of the corresponding DMSO (0.1%),were added to the wells. The cultures were maintained at 37 °C for 48 h. The cells were then collected by trypsinization, and the viable cells were counted by trypan blue exclusion with a hemocytometer. The percentage of viable cells was calculated using the formula [(Total cells counts—Dead cells counts)/Total cells counts] × 100. Cellular morphology was examined under an inverted light microscope with a 10× objective lens (Olympus).

### 2.8. Scratch Wound Assays

The A549 cells were added to six-well plates. Once the cells reached 70% confluence, the monolayer was scratched with a sterile tip (10 μL). The extracts of the aerial parts and bulbs, DMSO, and Taxol were then added to the wells for A549. After 24 or 48 h, scratch healing was recorded using an inverted microscope.

### 2.9. Invasion Assay

Cell invasion assays were performed using the Trans well chamber invasion assay (Merck Cat. no. ECM550). Briefly, A549 cells at a density of 6 × 10^5^ were added to 300 µL of prepared serum-free medium cell suspension for each insert. For the aerial part, bulb extracts were prepared (1.5, 6, and 12 µg/mL) in 500 µL media containing 10% fetal bovine serum and were added to the lower chamber. The cultures were incubated for 48 h in a cell culture incubator, and the cells were stained on the lower surface of the membrane. Any invasive cells present were stained. Cell invasion was observed under the inverted microscope and photographed (10×). The stained cells were then solubilized and measured at OD 560 nm in a plate reader.

### 2.10. Determination of Total Phenolic Content (TPC)

The total phenolic content (TPC) of the extracts was determined using the conventional Folin–Ciocalteu’s method as outlined by Singleton et al. [17]. Briefly, 20 μL of the extract was mixed with 400 μL of 0.2 N Folin–Ciocalteu’s reagent in a test tube, followed by the addition of 680 μL of distilled water. After a 3 min incubation period, 400 μL of 10% sodium carbonate solution was added, and the mixture was further incubated for 2 h at room temperature. Subsequently, the absorbance was measured at 760 nm using a spectrophotometer (Thermo Scientific Evolution TM 201, UV-VIS Spectrophotometer, USA). Varying concentrations of gallic acid ranging from 0.50 to 0.015625 mg/mL were employed to establish the standard curve. Plotting the absorbance values against the respective concentrations of gallic acid facilitated the determination of the phenolic content expressed as gallic acid equivalent (GAE) per gram of dry sample.

### 2.11. Determination of Total Flavonoid Content (TFC)

The total flavonoid content (TFC) in each extract was determined by employing a spectrophotometric assay, following the methodology outlined by Fukumoto et al. [18]. Each extract (250 μL) was combined with 2.15 mL of absolute methanol (Merck, 106009), along with the addition of 50 μL of 10% aluminum nitrate (Sigma Aldrich, 237973) and 50 μL of 1 M ammonium acetate. The mixture was incubated at room temperature for 40 min, and the absorbance was measured at 415 nm. A standard curve was constructed utilizing various concentrations of quercetin (ranging from 0.5 to 0.015625 mg/mL). Plotting the absorbance values against the respective concentrations of quercetin facilitated the determination of the flavonoid content expressed as quercetin equivalent per gram of dry sample.

### 2.12. Determination of Iron (III) Reducing/Antioxidant Power 

The ferric reducing/antioxidant power (FRAP) assay was employed to evaluate the total antioxidant capacity of the samples. The freshly prepared FRAP reagent was created by blending solutions of 300 mM pH 3.6 sodium acetate buffer, 10 mM TPTZ, and 20 mM FeCl_3_ (Carlo Erba, Italy, 451695) in a ratio of 10:1:1. Subsequently, 1.5 mL of the FRAP reagent was combined with 0.05 mL of the sample within a test tube, following the protocol outlined by Benzie et al. [19]. The mixture incubation was at 37 °C for 4 min, following which the absorbance was measured at 595 nm. Concentrations ranging from 31.25 to 1000 μM of FeSO_4_·7H_2_O (Merck, 103965) were used to construct the standard curve. The results were reported as FeSO_4_·7H_2_O equivalent antioxidant power.

### 2.13. Determination of DPPH Radical Scavenging Activity

The free radical scavenging activity of 2,2-diphenyl-1-picrylhydrazyl was measured using the spectrophotometric test method [20]. Equal volumes (750 μL) of the plant extract were mixed with 0.10 mM methanolic DPPH solution. Following 45 min incubation, absorbance was read at 517 nm. The result was calculated as SC_50_, defined as the sample concentration that causes a 50% drop in the DPPH radical concentration, with lower SC_50_ values indicating more radical scavenging activity. The SC_50_ value is defined as the sample concentration causing a 50% reduction in the DPPH solution and is calculated by graphing the values obtained from five or six different extract concentrations as mg/mL.

### 2.14. Phenolic Composition Analysis by HPLC-PDA

Phenolic composition analysis was carried out using an RP-HPLC system (Shimadzu Corporation LC 20AT, Kyoto, Japan) coupled with a photodiode-array (PDA) detector using the validated method with 25 phenolic standards [21]. The sample was first enriched with phenolic compounds by liquid–liquid extraction and then loaded into the device [22]. Initially, 10 mL of the sample extracts were evaporated using a rotary evaporator at 40 °C to remove the solvent. Next, 10 mL of purified water was added, and the pH was adjusted to 2 using concentrated HCl. The organic layers obtained from three extractions with diethyl ether and ethyl acetate were then combined. After the solvents were fully evaporated, the remaining residue was dissolved in 2 mL of methanol, filtered through a 0.45 μm membrane filter (RC membrane, 0.45 μm), and injected into the instrument for phenolic analysis.

In this method, acetonitrile, water, and acetic acid were used for the mobile phase by applying a programmed gradient. The mobile phase consisted of (A) 2% acetic acid in water and (B) acetonitrile:water (70:30). The volumes of the samples/standard injection were 20 µL, the column temperature was 30 °C, and the flow rate was 1.0 mL/min [23].

### 2.15. Statistical Analysis

Statistical analysis was performed using Student’s unpaired *t*-test on GraphPad. Data are presented as mean ± SD for at least three independently performed experiments.

## 3. Results and Discussion

### 3.1. Phenolic Properties and Antioxidant Capacities

Table 1 shows the total phenolic and total flavonoid contents of the methanolic extracts. Both contents were higher in the aerial part than in the bulb. In studies involving plant extracts, methanol and ethanol are commonly used as extraction solvents. Methanol was preferred in this study due to its higher polarity compared to ethanol, making it a more effective solvent for the extraction of bioactive compounds from plants. Its superior solvating capacity for polar compounds enables more efficient extraction of a wider range of phytochemicals, thus enhancing the overall yield of the desired bioactive constituents. However, in cell culture studies, after the complete evaporation of methanol, the solid extract was dissolved in diluted DMSO before use. Polyphenols, as vital secondary metabolites in plants, play a crucial role in various biological activities [24]. These compounds fall into two significant subclasses, phenolic acids and flavonoids, with numerous subcategories within each. Notably, nearly half of the phenolic compounds found in the aerial part in the present study consisted of flavonoids. The assessment of antioxidant capacity using two distinct methods demonstrated noteworthy findings. The FRAP value serves as a pivotal indicator of the overall antioxidant activity. In this study, the aerial part exhibited a notably superior antioxidant capacity.

In contrast to high FRAP values, lower DPPH values indicate a higher radical scavenging activity as well as a greater antioxidant capacity. The aerial part of the plant exhibited a very high radical scavenging ability compared to the bulb in this study. The heightened antioxidant capacity observed in the aerial part may be attributed to its rich potent antioxidant substance content, particularly polyphenols. The substantial presence of phenolic compounds in the aerial part strongly substantiates this observation. Similarly to the present investigation, various species of *Ornithogalum* L. have been shown to exhibit elevated polyphenol content and superior antioxidant capacity within their aerial segments compared to other anatomical parts [25].

The phenolic composition of both parts of the extracts was analyzed using HPLC-PDA. The data, categorized into two classes, phenolic acids and flavonoids, are outlined in Table 2. Twenty-five phenolic standards were used, and chlorogenic acid was identified as the major phenolic acid in the aerial part of the plant. The values for other phenolic acids, such as ferulic acid, protocatechuic acid, p-coumaric acid, and *p-*OH-benzoic acid, were also higher in the aerial part than in the bulb. Caffeic acid was detected in equal amounts in both parts. Quercetin was detected only in the aerial part, while chrysin was identified in both parts. Studies of different *Ornithogalum* spp. have reported that the total amount of phenolic substances varied between 2.20 and 11.00 mg GAE/g extract [25,26,27]. Consistent with the present study, research conducted with HPLC-UV on different *Ornithogalum* species has reported that these were rich in protocatechuic acid, coumaric acid, and gallic acid [25,26,27,28].

### 3.2. The Effects of the Extracts on the Morphology of A549 and H209 Cells

**Figure 1:**
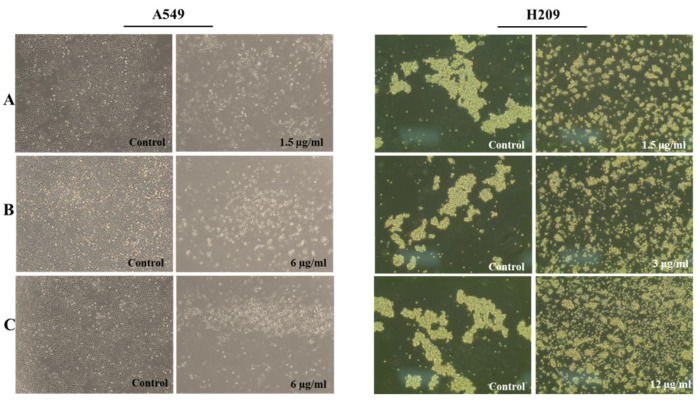
Cells’ morphological changes were observed under a microscope (magnification, ×4). A549 and H209 cells were treated different extracts at concentration (≤12 µg/mL) for 48 h. (**A**) Aerial parts, (**B**) B 1, and (**C**) B 2.

Lung cancer stands as one of the most prevalent malignancies globally, posing a significant human health challenge [29]. Lung cancer is generally divided into two categories: non-small-cell lung cancer (NSCLC) and small-cell lung cancer (SCLC). Specifically, A549 cells represent adherent NSCLC, while H209 cells typify suspense SCLC. H209 cells also exhibit metastatic potential. Figure 1 indicates the alterations in the morphology of the H209 and A549 cell lines seen after treatment with the extracts. The morphological changes in the treated cells were detected using an inverted microscope. No morphological changes were found in the cells treated with controls. Exposure of the cells to the aerial parts and bulb extracts caused morphological alterations in H209 and A549 cells. In particular, extracts of the aerial parts and bulb caused morphological alterations and detachment of the H209 and A549 cells at lower concentrations (≤12 µg/mL). A previous study reported that MeOH and ethyl acetate extracts of *O. balansae* flowers and bulbs exhibited inhibitory effects on different cancer cells [16].

### 3.3. Cell Growth Inhibition by Extracts Using the MTT Method

**Figure 2:**
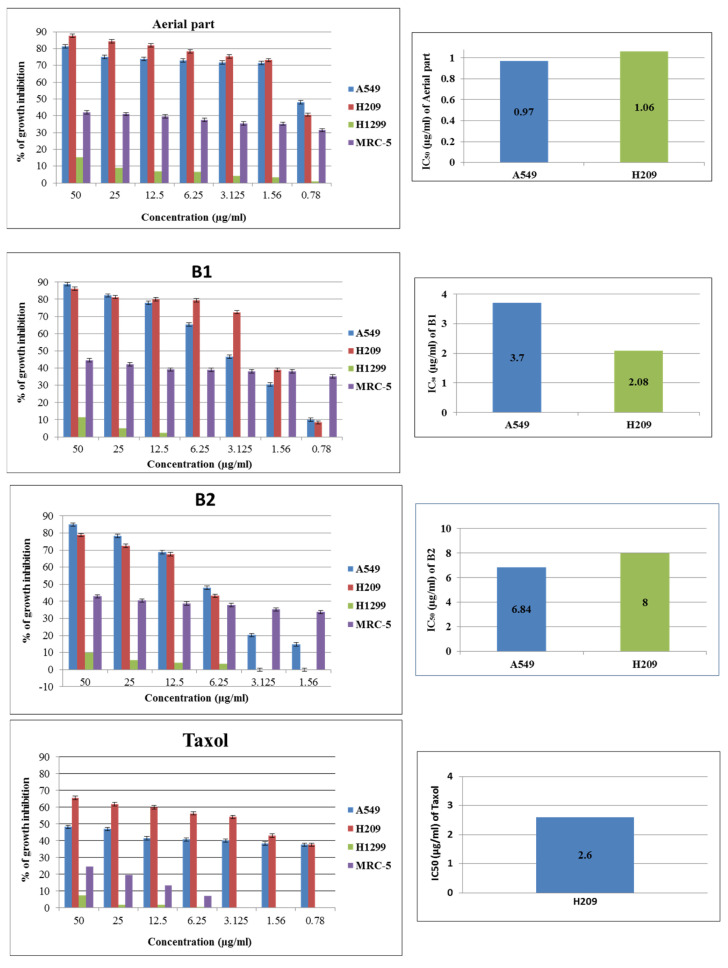
The growth inhibition of A549, H1299, H209, and MRC-5 cells after 48 h treatment with extracts and IC_50_ value. Results are expressed as mean ± SD from three independent experiments. Extracts induced statistically significant differences A549 and H209 cells (*p* < 0.0001).

**Figure 3:**
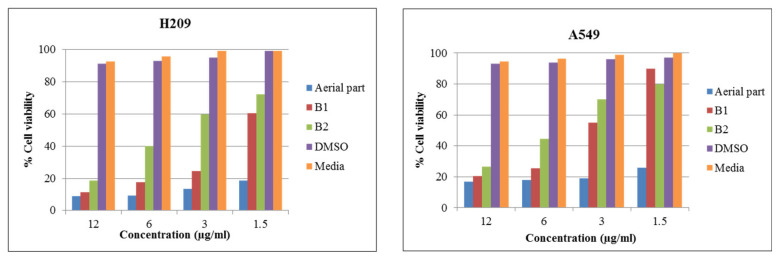
A549 and H209 cells were treated with extracts and negative control (DMSO, media) for 48 h. Cell viability was calculated by trypan blue assay. Results were repeated at least three times. Data are expressed as mean  ±  SD (n  =  3). *p* < 0.001 vs. negative control group (DMSO and media).

The primary objective of this study was to explore the inhibitory effects of extracts, particularly on diverse lung cancer cell lines. In contrast to prior research, this study specifically examined the growth inhibition effects of aerial part and bulb extracts on distinct lung cancer cell lines. Three cell lines and lung fibroblast cells were selected to investigate the effects of *O. balansae* extracts on cell lines. The MTT assays were employed to examine the effects of different concentrations on the anti-tumor activity of the extract against various lung cancer and fibroblast cells, namely A549, H209, H1299, and MRC-5. The anti-proliferative effects and cell viability results are shown in Figure 2. The extract derived from the aerial part demonstrated inhibitory effects exclusively on A549 and H209 cells, with no observable impact on H1299 lung cancer cells. The aerial part extracts induced 71.43 ± 0.63 and 73.16 ± 0.87% growth inhibition in A549 and H209 cells, respectively, at a concentration of 1.56 μg/mL (*p* ˂ 0.0001). According to the trypan blue dye assay results, A549 and H209 cell viability percentages were 24.40% and 18.4% at 1.50 μg/mL, respectively. However, this extract reduced cell viability only by 36.96% in the non-transformed MRC-5 cells. In terms of cytotoxicity, the IC_50_ values from aerial part extracts revealed significant differences between A549 and H209, with values of 0.97 ± 0.04 and 1.06 ± 0.07 μg/mL, respectively (Figure 2). The bulb analysis results showed that the IC_50_ values for these cells were 3.70 ± 0.47 and 2.08 ± 0.13 µg/mL for B1 and 6.84 ± 0.14 and 8.0 ± 0.31 µg/mL for B2, respectively. As depicted in Figure 2 and Figure 3, the aerial part significantly diminished the viability of both cells. The MTT assay was conducted to assess cell viability, and concurrent observations of alterations in cell morphology were made. The results from both cell viability and morphology assessments indicated a comparable impact between the aerial parts and bulbs. In addition, the positive control Taxol was demonstrated in H209 cells, with an IC_50_ value of 2.6 ± 0.27 µg/mL. *Ornithogalum* plants have been reported to exert anti-proliferative effects against a wide range of cancer cells, including leukemia [7,8,30], colorectal cancer [9], pancreas–brain–ovary [31], lymph node [30], and lung cancers [7,8,10]. A number of steroidal glycosides such as cardenolide, cholestane, spirostane, and stigmastane were isolated from *Ornithogalum* plants [32]. In certain studies conducted with cancer cell cultures, these glycosides have been shown to induce significant cytotoxicity at low concentrations compared to healthy cells. Overall, the findings indicate that the cholestane saponin exhibits cytotoxicity, particularly against cancer cells, without significant impact on normal cells. Moreover, its potency surpasses that of other anti-cancer agents such as doxorubicin, camptothecin, and paclitaxel [9,10]. In the present investigation, IC_50_ values for the extracts from both aerial parts and bulbs could not be determined in fibroblast MRC-5 cells. According to MTT assay results, the extracts showed less than 44.65% inhibition on MRC-5 cells.

In contrast to previous research, we hypothesized that the crude extract might induce a mild cytotoxic effect on normal cells, possibly attributed to the utilization of the crude extract [7,8,10,30].

### 3.4. Cell Viability of Extracts Using Trypan Blue

As shown in Figure 3, the bulb extracts B1 and B2 exhibited an inhibitory effect on H209 cells. The inhibition effect showed that the B1 extract possessed significant anti-proliferative activity on H209 cells at 24.47% cell viability at 3.0 μg/mL. At 6.0 µg/mL, these extracts displayed anti-tumor activity, causing a decrease greater than 70% in both A549 and H209 cells. Furthermore, the B2 extract demonstrated significant anti-tumor activity at 12.0 µg/mL against A549 and H209 cells. These extracts induced 73.59% and 81.47% growth inhibition in A549 and H209 cells, respectively. In addition, the aerial part extract produced 25.64% and 18.49% cell viability on A549 and H209 cells at the lowest dose of 1.50 μg/mL (*p* < 0.001).

### 3.5. Migration Ability of Extracts on A549 Cell

**Figure 4:**
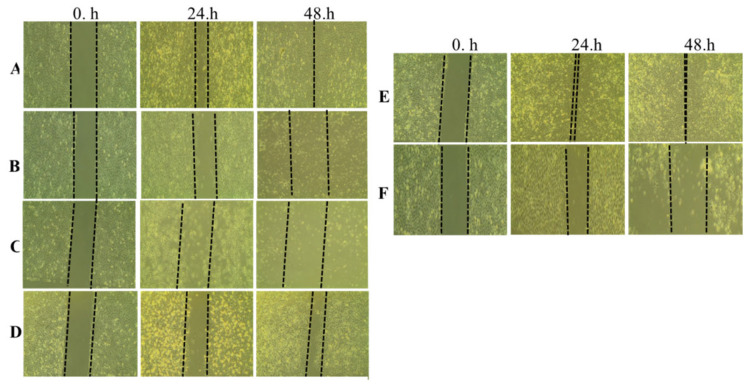
The demonstrated effect of aerial parts and bulbs extract, Taxol (positive control), and control (negative control, DMSO or water) on A549 cells motility. Cell motility was observed and photographed 24 and 48 h after creation of gap, using inverted microscope at 4 × magnification. (**A**) Control (DMSO), (**B**) B1 6 µg/mL, (**C**) aerial part 1.50 µg/mL, (**D**) Taxol 12 µg/mL, (**E**) control (water), and (**F**) B2 12 µg/mL.

Wound-healing assays were performed to evaluate the effect of the extracts on the migratory ability of A549 cells. In order observe the effect of extracts on lung cancer cell proliferation in vitro, a single scratch was made on A549 cells, and images of the scratched area were captured. As shown in Figure 4, the A549 cells were treated with the aerial part (1.50 µg/mL), B1 (6 µg/mL), B2 (12 µg/mL), Taxol as a positive control (12 µg/mL), and with negative controls (DMSO and water). Cell migration was observed under a microscope (magnification ×10) at 24 and 48 h. The results suggest that the extracts were capable of inhibiting A549 cell migration better than the controls. Metastasis is closely associated with the migration and invasion of cancer cells. The migration and invasion of lung cancer are critical factors in treatment [33,34]. The wound-healing assay was performed to determine whether extracts could suppress the motility of A549 cells. The results are shown in Figure 4, with 1.50 µg/mL of the aerial part and 6 µg/mL of B1 and 12 µg/mL of B2 markedly inhibiting A549 cell migration for 48 h. At the same time, degradation was observed in the A549 cells compared with control cells. Research has reported that steroidal glycosides inhibited the migration of triple-negative breast cancer and hepatocellular carcinoma [35,36].

### 3.6. Invasion Ability of Extracts on A549 Cell

**Figure 5:**
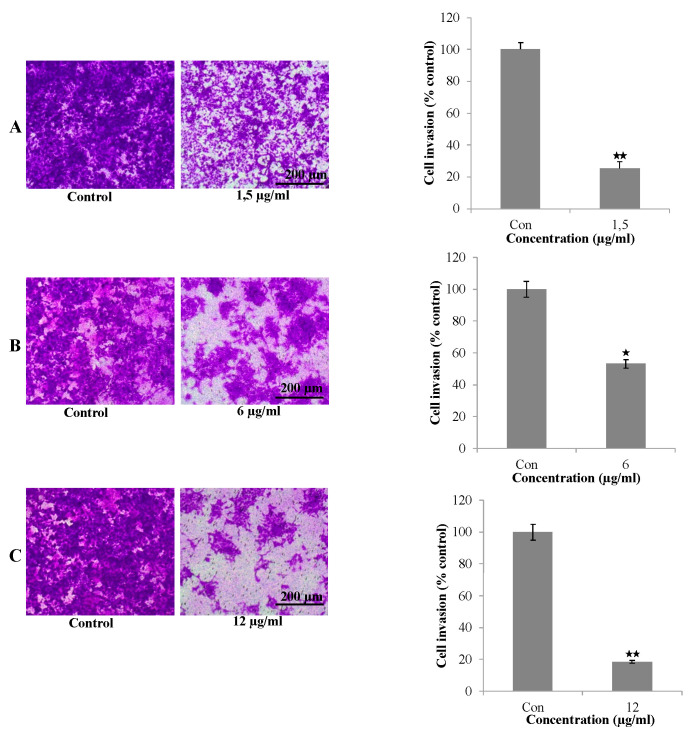
The invasion ability of A549 cells was investigated using invasion chamber assay. A549 cells were treated with (**A**) aerial part, (**B**) B1, and (**C**) B2, followed by QCM ECMatrix Cell Invasion Assay (8 µm). Results were analyzed using Student’s unpaired *t*-test in GraphPad (n = 3). * *p* < 0.005; ** *p* < 0.001. Con, control.

Invasion is another process entailing cancer cell proliferation and metastasis [37]. Invasion chamber assays were performed to evaluate the effect of the aerial part and B1 and B2 extracts on the invasive ability of A549 cells. As shown in Figure 5, 1.50 µg/mL of the aerial part, 6 µg/mL of B1, and 12 µg/mL of B2 significantly inhibited the invasion of A549 cells (*p* < 0.001). The number of invading cells decreased significantly compared with the untreated controls Consistent with the results of the wound-healing assay, the extracts also inhibited the invasive ability of A549 cells (* *p* < 0.005 and ** *p* < 0.001). The results indicate that the invasive ability of A549 cells was inhibited by the aerial parts and bulbs. Triple-negative breast cancer has also been shown to inhibit the invasion of steroidal glycosides [35]. In conclusion, the findings of the present study revealed both anti-proliferation for A549 and H209 (SCLC, metastatic) and anti-motility and anti-invasive effects of extracts of *O. balansae* against A549 human NSCLC cells. 

## 4. Conclusions

This comprehensive study sheds useful light on the promising inhibitory effects of *O. balansae* extracts against various lung cancer cell lines. The findings revealed distinct growth inhibition on NSCLC (A549) and metastatic (H209) lung cancer cells by the aerial part extracts, attributing their potent cytotoxicity to their composition of rich bioactive compounds, particularly polyphenols. The evidence of migratory and invasive capabilities of A549 cells being suppressed by these extracts further underscores their potential as therapeutic candidates against lung cancer metastasis. These observations align with previous research on the genus *Ornithogalum* and emphasize the significant potential of *O. balansae* extracts as effective agents against lung cancer proliferation and metastasis. Nonetheless, further investigations including in vivo studies and detailed mechanistic elucidation are now needed to validate and comprehend the full therapeutic scope of these extracts for the treatment of lung cancer.

## Figures and Tables

**Table 1:** Total Phenolics and Antioxidant Properties of the *O. balansae* Methanolic Extracts.

	Aerial Part	Bulb
Total Phenolic Content (mg GAE/100 g)	836.20 ± 28.34 ^a^	136.05 ± 5.28 ^b^
Total Flavonoid Content (mg QUE/100 g)	441.00 ± 12.30 ^a^	26.30 ± 1.20 ^b^
Total Antioxidant Capacity (FRAP) (µmol FeSO_4_·7H_2_O/g)	83.44 ± 5.80	6.45 ± 0.48
DPPH (SC_50_) (mg/mL)	0.43 ± 0.02 ^a^	66.15 ± 0.45 ^b^

^a^ Aerial part, ^b^ Bulb.

**Table 2:** Phenolic composition of the *O. balansae*.

Phenolic Acids		
	Aerial Part	Bulb
Gallic acid	-	6.90
Protocatechuic acid	22.29	-
Chlorogenic acid	413.54	-
*p*-OH Benzoic acid	14.63	-
*m*-OH Benzoic acid	-	-
Caffeic acid	6.71	6.08
Syringic acid	-	-
*p*-Coumaric acid	16.44	9.96
Ferulic acid	84.44	22.48
Ellagic acid	-	-
*t*-Cinnamic acid	-	2.11
**Flavonoids**		
	**Aerial Part**	**Bulb**
Rutin	-	-
Quercetin	27.77	-
Myricetin	-	-
Epicatechin	-	-
Daidzein	-	-
Luteolin	-	-
Chyrisin	5.07	1.15
Apigenin	-	-
Hesperetin	-	-
Rhamnetin	-	-
Pinocembrin	-	-
CAPE	-	-
Resveratrol		
Curcumin	-	-

(-): Not detected

## Data Availability

The data that support the findings of this study are available on request from the corresponding author. The data are not publicly available due to privacy or ethical restrictions.
